# A pan-cancer analysis indicates long noncoding RNA HAND2-AS1 as a potential prognostic, immunomodulatory and therapeutic biomarker in various cancers including colorectal adenocarcinoma

**DOI:** 10.1186/s12935-023-03163-7

**Published:** 2023-12-02

**Authors:** Pouria Samadi, Mina Shahnazari, Abolfazl Shekari, Fatemeh Maghool, Akram Jalali

**Affiliations:** 1 Research Center for Molecular Medicine, Hamadan University of Medical Sciences, Hamadan, Iran; 2 Student Research Committee, Hamadan University of Medical Sciences, Hamadan, Iran; 3 Department of Genetics and Molecular Medicine, School of Medicine, Zanjan University of Medical Sciences, Zanjan, Iran; 4 Poursina Hakim Digestive Diseases Research Center, Isfahan University of Medical Sciences, Isfahan, Iran

**Keywords:** HAND2-AS1, Pan-cancer, Prognostic biomarker, Immunotherapy, WGCNA

## Abstract

The HAND2-AS1 (HAND2 Antisense RNA 1) Long noncoding RNA (lncRNA) has emerged as a participant in the initiation of various cancer types, underscoring its pivotal involvement in both oncological processes and immune responses. To gain deeper insights into the functional nuances of HAND2-AS1 and identify novel avenues for cancer immunotherapy, a comprehensive evaluation of this gene was undertaken. Here, based on the co-expression network analysis and construction of interacting lncRNA–mRNA genes, we introduce the HAND2-AS1 lncRNA, emphasizing its key roles in tumorigenesis and immune regulation. Our study spans across 33 distinct cancer types, revealing the HAND2-AS1’s aberrant expression patterns, methylation variations, mutational signatures, and immune engagement. Across a majority of tumors, HAND2-AS1 exhibited a propensity for down-regulation, remarkably an association with poor survival outcomes. The outcomes of functional enrichment analyses strongly suggest HAND2-AS1’s engagement in tumor progression and its association with various immune pathways across diverse tumor classifications. Additionally, a positive correlation emerged between HAND2-AS1 expression and the infiltration levels of key immune cells, encompassing not only immunosuppressive entities such as tumor-associated macrophages, cancer-associated fibroblasts, and Tregs, but also immune effector cells like NK cells and CD8+ T cells, spanning a pan-cancer context. Furthermore, the differential expression of HAND2-AS1 appears to have downstream consequences on various pathways, thus implicating it as a potential regulator in diverse cancer types. Finally, we have employed CRC tumor and normal samples to carry out clinical validation of HAND2-AS1. Our study unveils HAND2-AS1’s potential as a pan-cancer tumor suppressor, and its essential role in the tumorigenesis and immune surveillance. The increased HAND2-AS1 expression emerges as a promising candidate for prognostic evaluation, therapeutic strategy, and a focal point for immunotherapeutic interventions.

**Supplementary Information:**

The online version contains supplementary material available at 10.1186/s12935-023-03163-7.

## Introduction

Colorectal cancer (CRC), recognized as one of the most prevalent malignant neoplasms globally, stands as the third foremost cause of cancer-related mortalities on a global scale. In the year 2020 alone, the incidence of new CRC cases exceeded 1.9 million, leading to a death toll of 930,000 cases [1]. The absence of potential diagnostic and highly specific biomarkers has leaded to the the late diagnosis of CRC, often at an advanced stage, resulting in a rather modest 5-year survival rate ranging from 40 to 60% [2]. Despite the significant advancements in anti-cancer therapies, contributing to an enhanced overall survival (OS) among CRC patients, the prognosis remains poor due to the persistently elevated recurrence and metastasis rates inherent to advanced stages of CRC [3]. Then, gaining insights into the molecular mechanisms underlying CRC is crucial for advancing early diagnosis, treatment, and prognosis. To achieve this, many studies has been conducted to investigate the role of long non-coding RNAs (lncRNAs) in modulating various biological processes of CRC cells through intricate lncRNA–mRNA regulatory networks. These studies aim to shed light on the initiation, progression, and metastasis of CRC, ultimately leading to improved strategies for early detection, effective treatment, and better patient outcomes [4–7].

Non-coding RNA (ncRNA) is a term used to describe a class of transcripts that do not have the ability to encode proteins and constitute over 98% of the entire genome transcript [8]. LncRNAs, are RNA molecules characterized by a length exceeding 200 nucleotides, and they do not possess the capacity to code for proteins [9]. While lncRNAs often possess poly-A tails similar to protein-encoding mRNAs, they do not undergo translation into proteins. Notably, lncRNAs exhibit higher tissue specificity compared to protein-encoding mRNAs, making them potentially valuable as biomarkers for various diseases [10].

HAND2-AS1 (HAND2 Antisense RNA 1) is an interesting lncRNA that exhibits an antisense orientation to HAND2, another gene situated on the same genomic locus of chromosome 4q33-34. Notably, these two genes are arranged head-to-head, with a shared promoter region that can activate their transcription concurrently. The interplay between HAND2-AS1 and HAND2 adds complexity to their functional regulation and suggests potential regulatory crosstalk [11, 12].

A notable aspect of HAND2-AS1 is its capability to undergo alternative splicing, giving rise to multiple distinct transcript variants. These alternative splicing events diversify the repertoire of HAND2-AS1 isoforms, potentially leading to a wide range of functional implications specifically across human cancers [13]. Understanding the therapeutic, prognostic and immune-related functions of HAND2-AS1 variants could shed light on its regulatory roles in different biological processes including pan-cancers.

In this research, we conducted an extensive analysis of RNA-seq data derived from the Cancer Genome Atlas (TCGA) and the Gene Expression Omnibus (GEO) database. The aim was to uncover pivotal lncRNAs and mRNAs with key roles in the context of CRC progression. By constructing a lncRNA–mRNA regulatory network involving these molecules, we aimed to elucidate the molecular mechanisms underlying CRC occurrence and progression. Furthermore, our goal was to uncover novel predictive markers for CRC development, providing valuable insights for clinical diagnosis and therapeutic strategies. In this regard, HAND2-AS1 as a hot lncRNAs with an important role in pathogenesis of many cancers has been comprehensively analyzed over cancers specifically colorectal adenocarcinoma.

## Materials and methods

### Design of the study and data processing approach

**Fig. 1:**
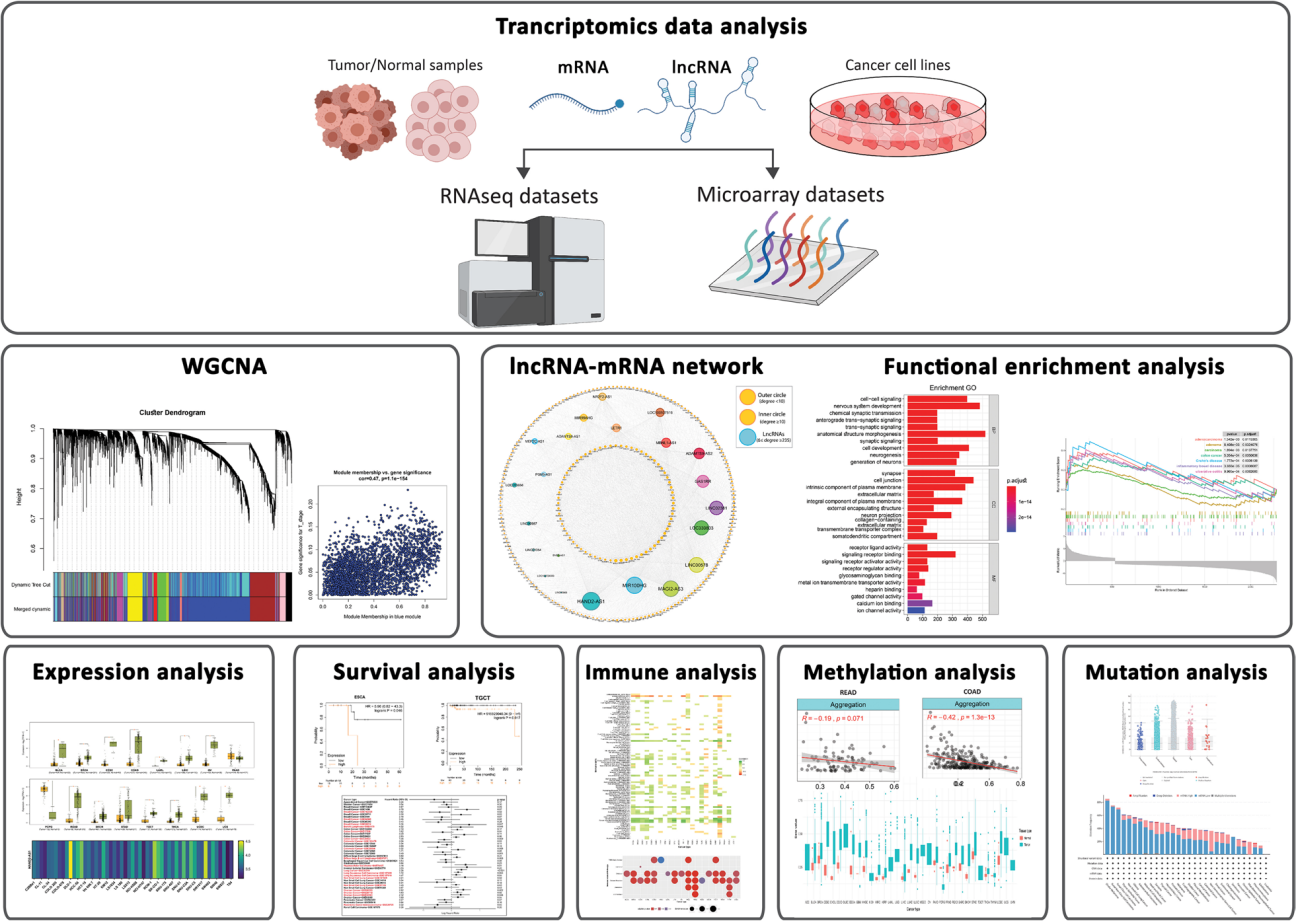
Two distinct collections of microarray and next-generation sequencing datasets were acquired and subjected to analysis in order to uncover differentially expressed genes (DEGs) and lncRNAs (DE-lncRNAs) across a range of cancers. Subsequently, a subset of robust interacting DEGs and DE-lncRNAs associated with TNM staging (blue module) were identified, forming the basis for subsequent regulatory network construction and functional enrichment analyses. Finally, a comprehensive series of downstream analyses was carried out, focusing on HAND2-AS1 as the most prominently interacting DE-lncRNA within the regulatory network

The schematic workflow representing the study’s design and the data collection is demonstrated in Fig. 1. For gene expression data acquisition, we obtained RNA-seq data along with corresponding clinical information from the TCGA database (https://portal.gdc.cancer.gov/), with a specifical focus on colon adenocarcinoma (COAD) and rectal adenocarcinoma (READ). The TCGAbiolinks package was employed for data retrieval and processing [14]. By employing DESeq2 package in R, we identified potential DEGs and differentially expressed lncRNAs (DE-lncRNAs) with enhanced robustness and reliability [15]. Ultimately, the DEGs and DE-lncRNAs from the RNA-Seq TCGA datasets were identified by applying the cut-off criteria of false discovery rate (FDR) < 0.05 and |absolute log2-fold change (FC)| > 1.

### Identification of the key DEGs/DE-lncRNAs interacting modules using WGCNA

For the selection of key co-expression modules comprising DEGs/DE-lncRNAs linked to TNM staging, we utilized the Weighted Gene Co-expression Network Analysis (WGCNA) algorithm implemented through the R package WGCNA [16]. A co-expression network was constructed using WGCNA, which calculated Pearson’s correlation coefficients (PCCs) across gene pairs, transforming them into an adjacency matrix with a soft threshold power (β = 3) to ensure a scale-free topology. Modules were identified by hierarchical clustering of DEGs/DE-lncRNAs, with a minimum module size set at 30 and a cut height of 0.3 for merging similar modules. Modules were then correlated with clinical traits, specifically TNM staging, to identify those with significant clinical relevance for further exploration of their biological functions.

### Identifying key targets via the construction of DEGs/DE-lncRNA regulatory network

Following the identification of the final module associated with TNM staging containing DEGs and DE-lncRNAs, we further explored the potential mRNA-lncRNA interactions within this module by employing PCCs analysis to assess the relationship between DE-lncRNAs and DEGs. Pairs with PCCs greater than 0.6 and an FDR less than 0.05 were considered as strong interactions. Finally, the interactions among DE-lncRNAs and DEGs were imported into Cytoscape 3.8.2 for the construction and visualization of the network.

### Functional enrichment analysis

To gain insights into the biological significance of the selected module containing interacting DEGs/DE-lncRNAs, functional enrichment analysis was carried out. The analysis involved utilizing the R package ClusterProfiler [17] to perform Gene Ontology (GO) and Kyoto Encyclopedia of Genes and Genomes (KEGG) enrichment [18]. For the GO enrichment analysis, ClusterProfiler was applied to generate a bar plot, depicting enriched pathways. In both the GO and KEGG analyses, terms with an adjusted p-value lower than 0.05 were considered statistically significant. Furthermore, the ClusterProfiler package was employed to conduct Gene Set Enrichment Analysis (GSEA), focusing on the genes targeted by HAND2-AS1. The GSEA was conducted using the REACTOME pathway database with the FDR cutoff of < 0.05.

### Evaluation of HAND2-AS1 expression level across cancers

To validate the expression levels of HAND2-AS1, we utilized GEPIA2, as a powerful tool for gene expression analysis [19]. The standardized calculation method from the UCSC Xena database [20] was employed in this analysis. Utilizing RNA-seq expression data obtained from the TCGA database, we conducted a comparative assessment of HAND2-AS1 expression levels across 33 distinct cancer tissue types and corresponding normal tissues obtained from the GTEx database. Additionally, we have used the GSE36133 dataset to select various CRC cell lines for in-depth analysis of HAND2-AS1 expression.

### The prognostic impact of HAND2-AS1 expression in diverse cancer types

We conducted a comprehensive investigation into the impact of HAND2-AS1 expression on patient survival across different cancer types using the Kaplan–Meier Plotter online tool (https://kmplot.com/analysis/) [21]. By employing the Kaplan–Meier survival analysis, we explored the complex relationship between HAND2-AS1 expression levels and two crucial survival metrics, called OS and recurrence-free survival (RFS), across distinct cancer types. The log-rank test was employed to evaluate the prognostic significance of HAND2-AS1. To further evaluate the predictive power of HAND2-AS1 expression in terms of patient outcomes, we performed univariate Cox regression analyses. This assessment encompassed pan-cancer data derived from various microarray studies, enabling us to predict OS, disease-free survival (DFS), progression-free survival (PFS), and RFS across diverse cancer types. The analysis was conducted utilizing the survival, survminer, and ggplot2 packages in the R programming environment.

### Immune infiltration and immune-related pathway analysis

We aimed to explore the potential association between HAND2-AS1 expression and the tumor microenvironment in pan-cancer by evaluating various metrics, including stromal score, ESTIMATE score, immune score, tumor purity, and immune-related pathways. In this case, we have employed multiple algorithms including XCELL, QUANTISEQ, CIBERSORT-ABS, EPIC and TIMER. To visualize the results effectively, we have used ggplot2 R package.

### DNA methylation analysis

Methylation data retrieved from the SMART App (http://www.bioinfo-zs.com/smartapp) was utilized to explore the association between HAND2-AS1 expression and methylation patterns in different tumors, with a specific focus on COAD and READ cancers, as per the TCGA project. The box plot visualizations were created using the ggplot2 package in R.

### Mutation profile analysis

Exploring the mutational landscape of HAND2-AS1 across pan-cancer entailed the utilization of the cBioPortal tool (http://www.cbioportal.org/). Specifically, we directed our analysis towards the “TCGA Pan-Cancer Atlas Studies” cohort for an encompassing investigation. Through this approach, we explored the frequency of genetic alterations, the types of mutations, and the range of copy number alterations (CNAs) impacting HAND2-AS1. Our investigation was precisely extended to encompass an analysis of the sites bearing mutations.

### Patients

We collected a complete set of 20 CRC tissue samples from Iranian patients who were referred to the Poursina Hakim Research Institute in Isfahan. These patients were diagnosed with CRC at different stages between 2021 and 2022. The study protocol was conducted in accordance with ethical standards and received approval from the ethics committee of Hamadan University of Medical Sciences, following the ethical code IR.UMSHA.REC.1402.396. Importantly, all participants included in the study had not undergone any prior treatments, including chemotherapy or radiotherapy, before the tissue samples were obtained. To serve as controls, adjacent healthy tissue samples were also collected alongside each tumor and inflammatory sample. All collected samples were meticulously stored at a temperature of − 80 °C until they were subsequently subjected to downstream expression analysis.

### Validation of HAND2-AS1 expression in CRC using real-time quantitative PCR assay

Tumor and normal tissues obtained from CRC patients underwent total RNA extraction using the RNX-Plus kit (CinnaGen, Iran). The extracted RNA was then reverse transcribed into complementary DNA (cDNA) using the RevertAid first-strand cDNA synthesis kit (Thermo Fisher Scientific, USA). Quantitative reverse transcription PCR (RT-qPCR) was conducted in duplicate for each sample. The Real-Time PCR Detection System LightCycler 96 (Roche, USA) and the SYBR Green method were employed according to the manufacturer’s instructions.

The RT-qPCR analysis involved designed primers targeting HAND2-AS1 as the gene of interest and GAPDH as the reference gene. The specific primer sequences used were as follows: HAND2-AS1 primers: Sense: 5′-GTGGCTGGTATCGGTGTTC-3′, Antisense: 5′-GTGGAGAGGACTGGTTTCG-3′ and GAPDH primers (Sinaclon, Iran): Sense: 5′-AAGGCTGTGGGCAAGGTCATC-3′, Antisense: 5′-GCGTCAAAGGTGGAGGAGTGG-3′. The relative gene expression levels of HAND2-AS1 and GAPDH were assessed using the 2^−ΔΔCt^ method, as outlined by Livak’s formula for fold change calculation. The expression levels were normalized with respect to the GAPDH mRNA levels. Moreover, to validate the findings, an independent clinical dataset with a larger sample size was utilized. Specifically, the GEO datasets GSE87211 (tumor = 202, normal = 157) and GSE68468 (tumor = 186, normal = 55), were also employed as external validation sets. This additional analysis strengthened the reliability and significance of our results.

### Statistical analysis

All the data presented in this paper are represented as the mean ± standard deviation (SD). Correlation analysis between the two variables was performed using Spearman’s or Pearson’s tests. The statistical analysis was carried out using R v4.2.2 and Prism v9.00. Additionally, a p-value < 0.05 was considered as the threshold for statistical significance.

## Results

### Identification of robust DEGs and DE-lncRNAs by differential expression analysis and WGCNA

The study workflow is constructed upon the data acquired from TCGA-COAD-READ and is divided into eight sequential steps, as illustrated in Fig. 1. Within the datasets, around 5600 DEGs and DE-lncRNAs were identified, with the number of down-regulated (DR) entities showing a relatively higher count compared to up-regulated (UR) DEGs and DE-lncRNAs (Additional file 1: Table S1).

**Fig. 2:**
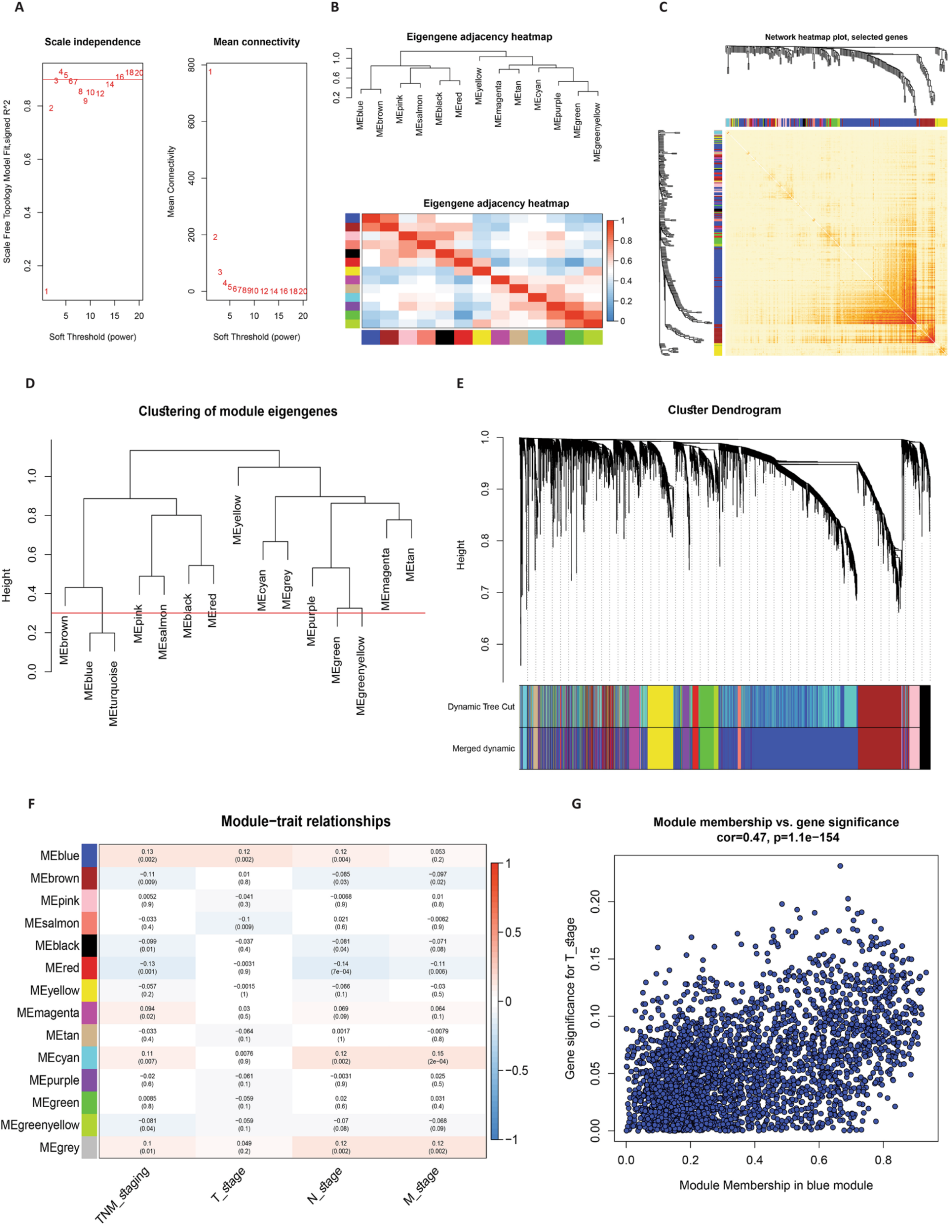
The identification of pivotal modules demonstrating substantial associations with TNM staging within the TCGA COAD-READ dataset is outlined through the subsequent steps: **A** evaluation of the scale-free fit index (displayed on the left) and mean connectivity (presented on the right) across varying soft-threshold powers. **B** Presentation of an eigengene adjacency heatmap. **C** Depiction of a network heatmap plot featuring the principal modules. **D** Clustering of module eigengenes, with a distinctive red line marking the cut height (set at 0.3). **E** Formation of clustering dendrograms, aligning robust DEGs and DE-lncRNAs with their corresponding modules based on a unique dissimilarity measure (1-TOM). **F** Generation of a heatmap illustrating the correlation between module eigengenes and clinical attributes, specifically TNM staging, within the context of colorectal cancer (CRC). Each cell contains both the p-value and the correlation coefficient. **G** Illustration of a scatter plot emphasizing the paramount significance of the key module (blue module) in relation to CRC’s TNM staging

To construct a co-expression network using the TCGA COAD-READ dataset, a stepwise approach was employed. The initial step involved the creation of a sample clustering tree to identify and exclude outlier samples lacking of biological relevance. Subsequently, a soft-thresholding power of β = 3 (resulting in a scale-free R2 = 0.9) was set, resulting in the identification of fourteen distinct modules characterized by varying colors with non-clustering DEGs and DE-lncRNAs were designated as gray module (Fig. 2A–E). Furthermore, module-trait relationships were assessed (Fig. 2F), revealing that the blue module demonstrated heightened significance concerning clinical attributes associated with TNM staging (Cor = 0.47/p = 1.1e−154) (Fig. 2G). Within this context, a total of 2299 interacting DEGs and DE-lncRNAs within the blue module were selected for further in-depth analysis (Additional file 1: Table S2).

### Identification of pivotal targets through analysis of the lncRNA–mRNA interacting network

**Fig. 3:**
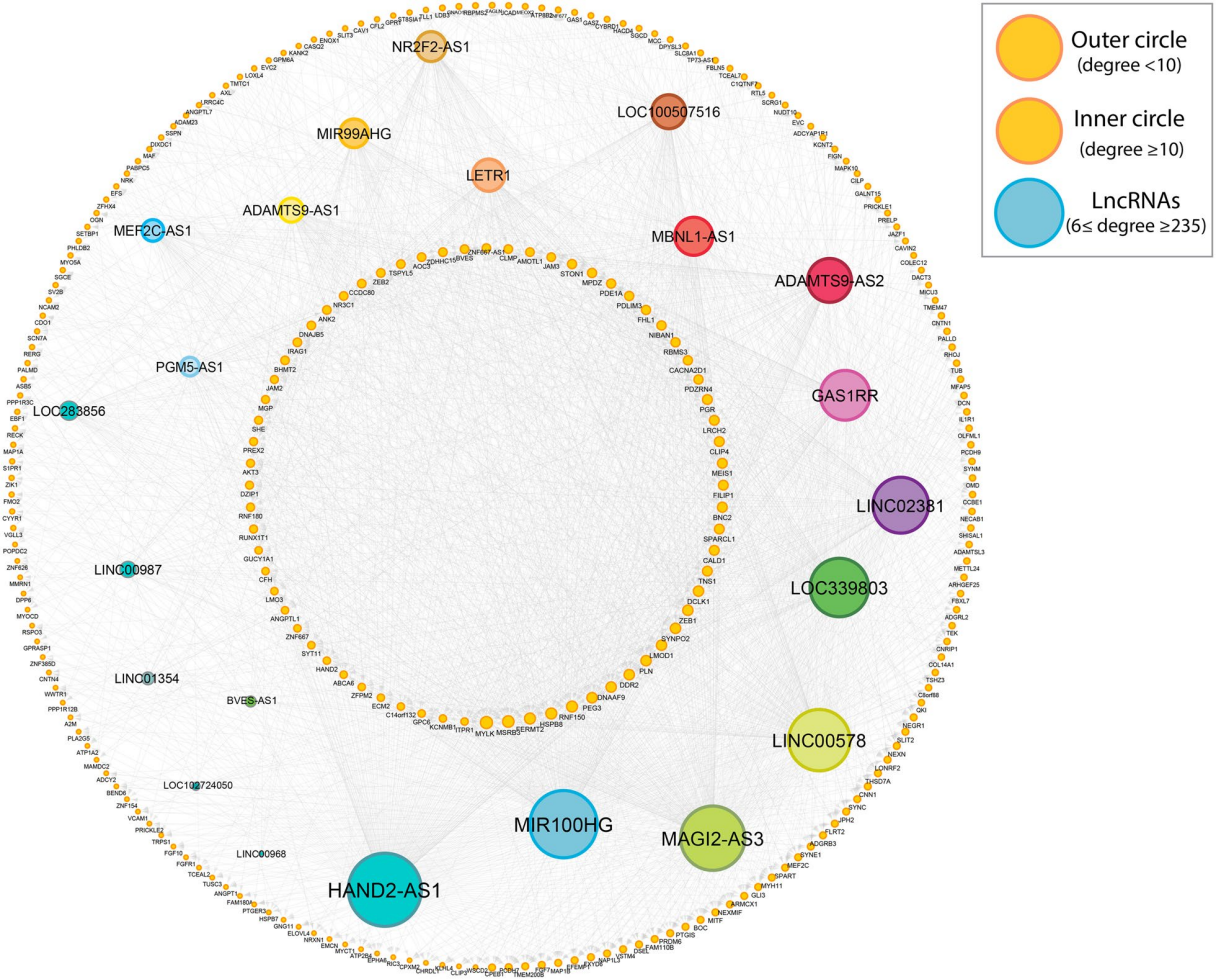
The regulatory network of lncRNA–mRNA interactions is constructed by considering significantly differentially expressed genes (DEGs) and DE-lncRNAs. Various colors are employed to distinguish distinct lncRNA molecules, with node sizes varying based on their degree, where larger nodes represent higher degrees. The outer circle encompasses mRNAs with degrees less than 10, while the inner circle represents mRNAs with degrees equal to or greater than 8

To construct a lncRNA–mRNA network, a total of highly correlated 604 DEGs and 22 DE-lncRNAs with potential interactions were used (Additional file 1: Table S3). Complex reciprocal interaction of different lncRNA–mRNA pairs has shown a higher number of edges (more than 100) shared by HAND2-AS1 (235 node), MIR100HG (225 node), MAGI2-AS3 (220 node), LINC00578 (179 node), LOC339803 (168 node), LINC02381 (132 node), GAS1RR (120 node) and ADAMTS9-AS2 (104 node) (Additional file 1: Table S4). This high level of connectivity may imply their significant involvement in the process of CRC carcinogenesis (Fig. 3). Ultimately, aligning with the aims of our investigation, we opted to focus on the HAND2-AS1 lncRNA due to its remarkable node connectivity, positioning it as a prime candidate for further downstream analyses.

### Functional and gene set enrichment analysis of HAND2-AS1

**Fig. 4:**
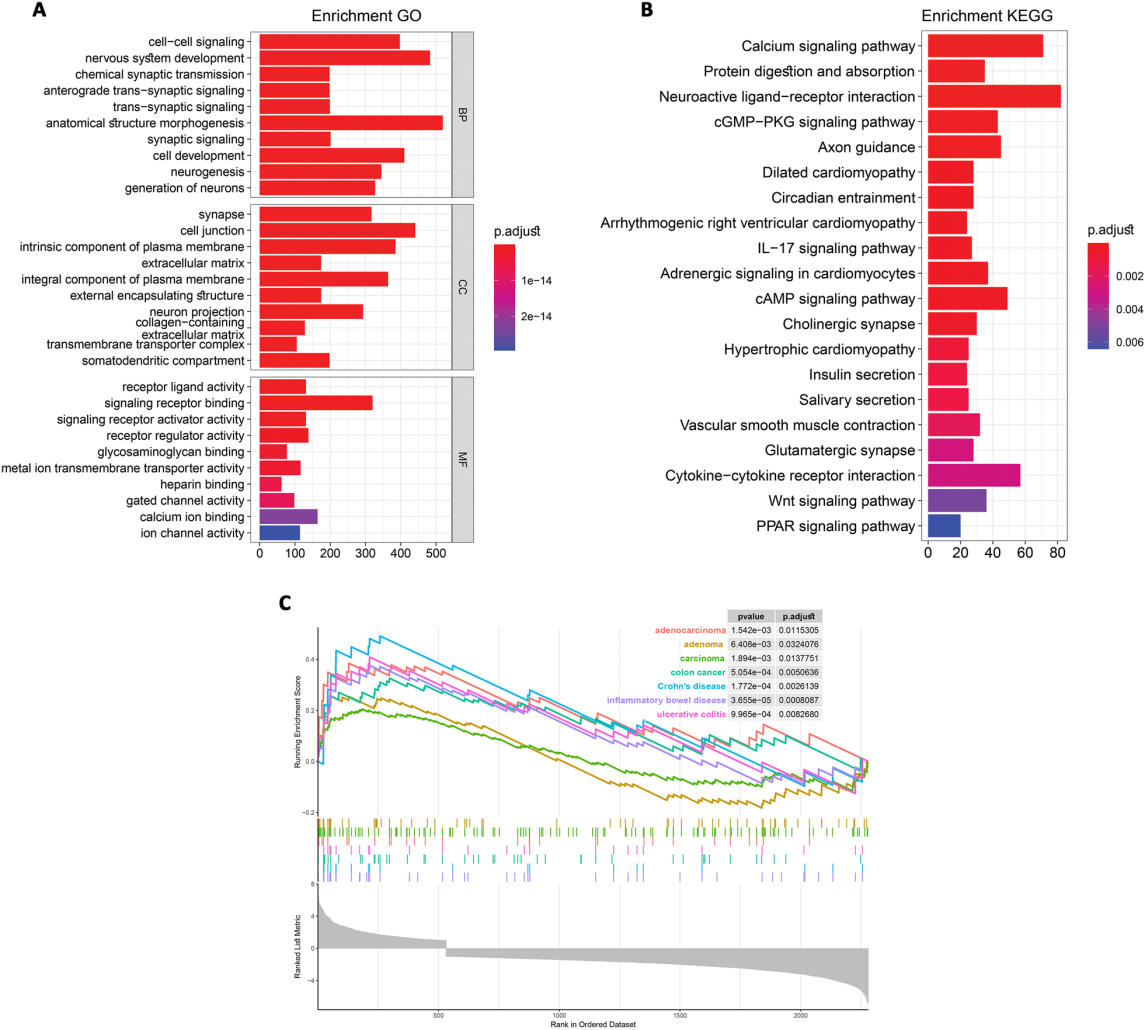
GO and KEGG pathway enrichment analyses and GSEA of blue module. **A** Results illustrating enriched biological processes, cellular components, and molecular functions are provided. The size of each circular node corresponds to the gene ratio of the enriched gene count. **B** KEGG pathway enrichment analyses reveal the pathways that are enriched. The size of circular nodes reflects the gene ratio of the enriched genes. **C** Moreover, a Gene Set Enrichment Analysis (GSEA) was conducted on the HAND2-AS1 targeted genes within the TCGA COAD-READ dataset

Functional enrichment analysis was executed by employing the DAVID platform through the clusterProfiler package in R. This facilitated the exploration of noteworthy terms in GO enrichment, encompassing biological process (BP), cellular component (CC), and molecular function (MF), as well as KEGG pathway analyses for both the blue module and HAND2-AS1 targeted DEGs individually. Specifically, for the DEGs within the blue module (Additional file 1: Table S5), the findings revealed prominent processes in the BP category, such as cell-cell signaling, nervous system development, chemical synaptic transmission, regulation of system processes, and regulation of localization, among others (Fig. 4A). In the category of CC, there were significant enrichments in terms like synapse, cell junction, intrinsic component of plasma membrane, extracellular matrix, integral component of plasma membrane, external encapsulating structure, cell projection, etc. (Fig. 4A). Furthermore, terms such as receptor ligand activity, signaling receptor binding, signaling receptor activator activity, and receptor regulator activity emerged as significantly enriched MF categories (Fig. 4A). Additionally, we conducted KEGG pathway enrichment analysis, revealing the top ten enriched terms, including the Calcium signaling pathway, Protein digestion and absorption, cGMP-PKG signaling pathway, IL-17 signaling pathway, cAMP signaling pathway, Cytokine-cytokine receptor interaction, and Wnt signaling pathway (Fig. 4B) (Additional file 1: Table S6).

**Fig. 5:**
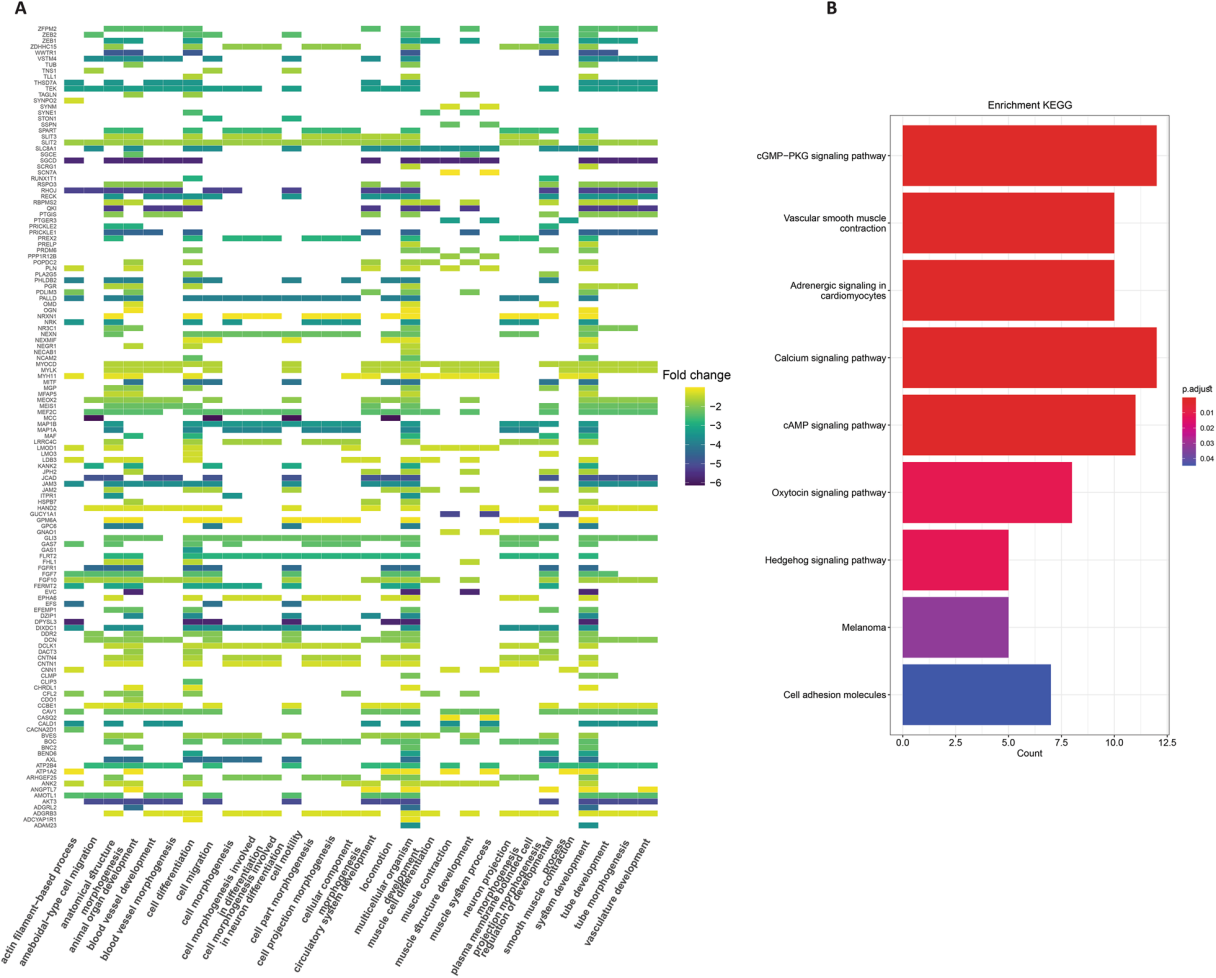
The investigation involved Gene Ontology (GO) and Kyoto Encyclopedia of Genes and Genomes (KEGG) pathway enrichment analyses, along with Gene Set Enrichment Analysis (GSEA) of genes targeted by HAND2-AS1. **A** The outcomes encompassed biological process, cellular component, and molecular function analyses. **B** Additionally, KEGG pathway enrichment analyses were carried out. The size of round nodes corresponded to the gene ratio of the enriched gene number

Furthermore, we conducted distinct GO and KEGG analyses focusing on the HAND2-AS1 targeted DEGs. These analyses revealed enrichment in pathways closely associated with cancer, as illustrated in Fig. 5A, B (Additional file 1: Tables S7, S8). In addition, to gain deeper insights into the biological functions of the HAND2-AS1 targeted DEGs, we employed the GSEA method based on the TCGA COAD-READ dataset. As depicted in Fig. 4C, the targeted DEGs exhibited remarkable enrichment across diverse pathways including adenocarcinoma, adenoma, carcinoma, colon cancer, Crohn’s disease, ulcerative colitis, and inflammatory bowel diseases, all with a significant FDR value of < 0.05.

### Expression analysis of HAND2-AS1 in pan-cancer and CRC cell lines

**Fig. 6:**
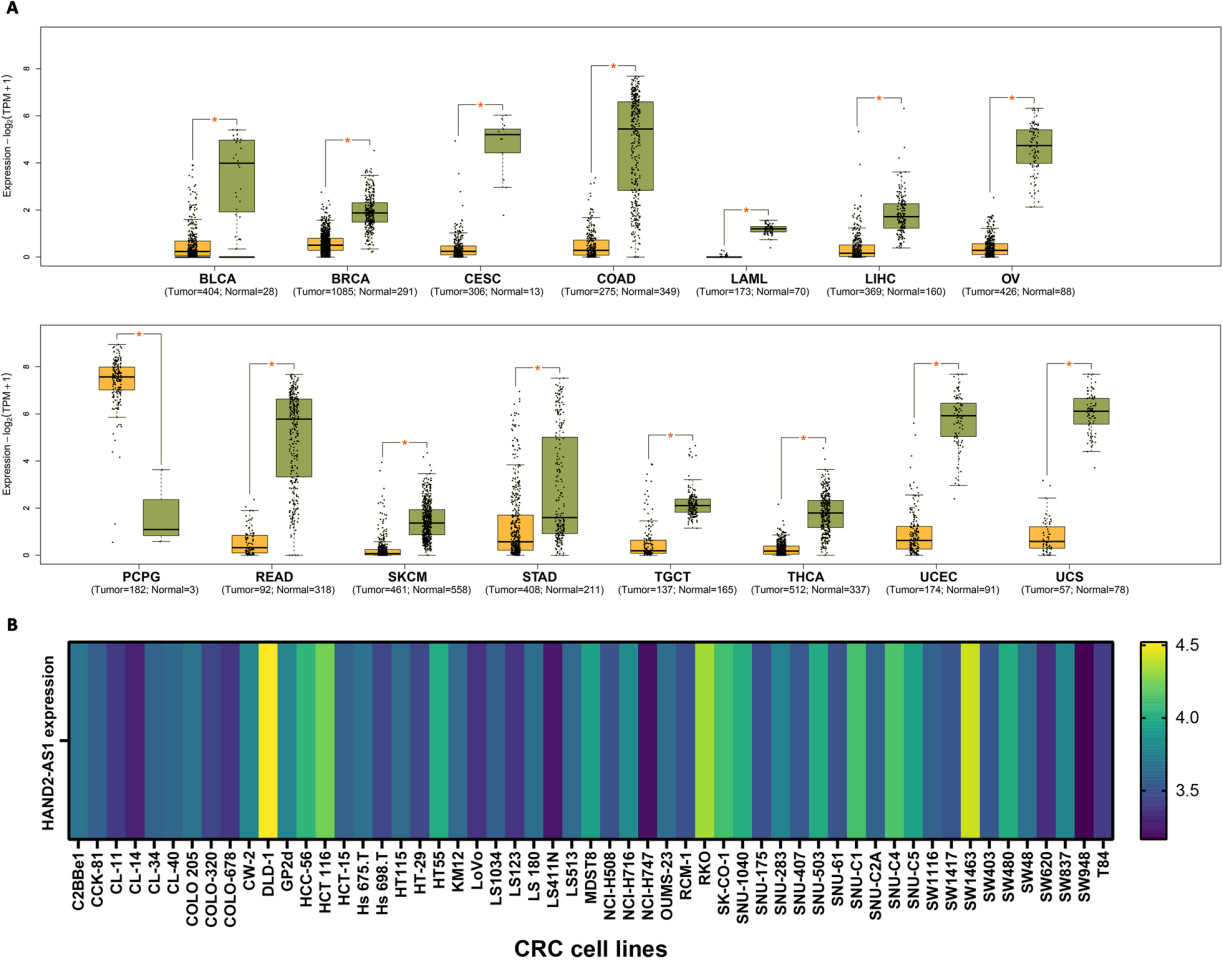
HAND2-AS1 expression levels exhibit variability across distinct cancer types. **A** Illustrates HAND2-AS1 level profiles between tumors and normal tissues, focusing on significant cancers. **B** Depicts the expression levels of HAND2-AS1 in diverse CRC cell lines. *p < 0.05

We examined the correlation between HAND2-AS1 expression in cancer tissues and normal tissues using datasets from TCGA and GTEx. Notably, HAND2-AS1 expression was found to be significantly lower in cancer tissues compared to normal tissues in various cancers, including but not limited to breast invasive carcinoma (BRCA), bladder urothelial carcinoma (BLCA), cervical squamous cell carcinoma and endocervical adenocarcinoma (CESC), COAD, liver hepatocellular carcinoma (LIHC), ovarian cystadenocarcinoma (OV), acute myeloid leukemia (LAML), pheochromocytoma and paraganglioma (PCPG), READ, stomach adenocarcinoma (STAD), skin cutaneous melanoma (SKCM), testicular germ cell tumors (TGCT), uterine corpus endometrial carcinoma (UCEC), thyroid carcinoma (THCA), and uterine carcinosarcoma (UCS), as depicted in Fig. 6A.

Additionally, we investigated HAND2-AS1 expression levels across different CRC cell lines. Notably, DLD-1, SW-1463, RKO, and HCT-116 exhibited comparatively higher expression levels, while SW-948, NCI-H747, LS411N, and CL-11 displayed lower expression levels in relation to the other cell lines (Fig. 6B).

### HAND2-AS1 prognostic value analysis in pan-cancer

**Fig. 7:**
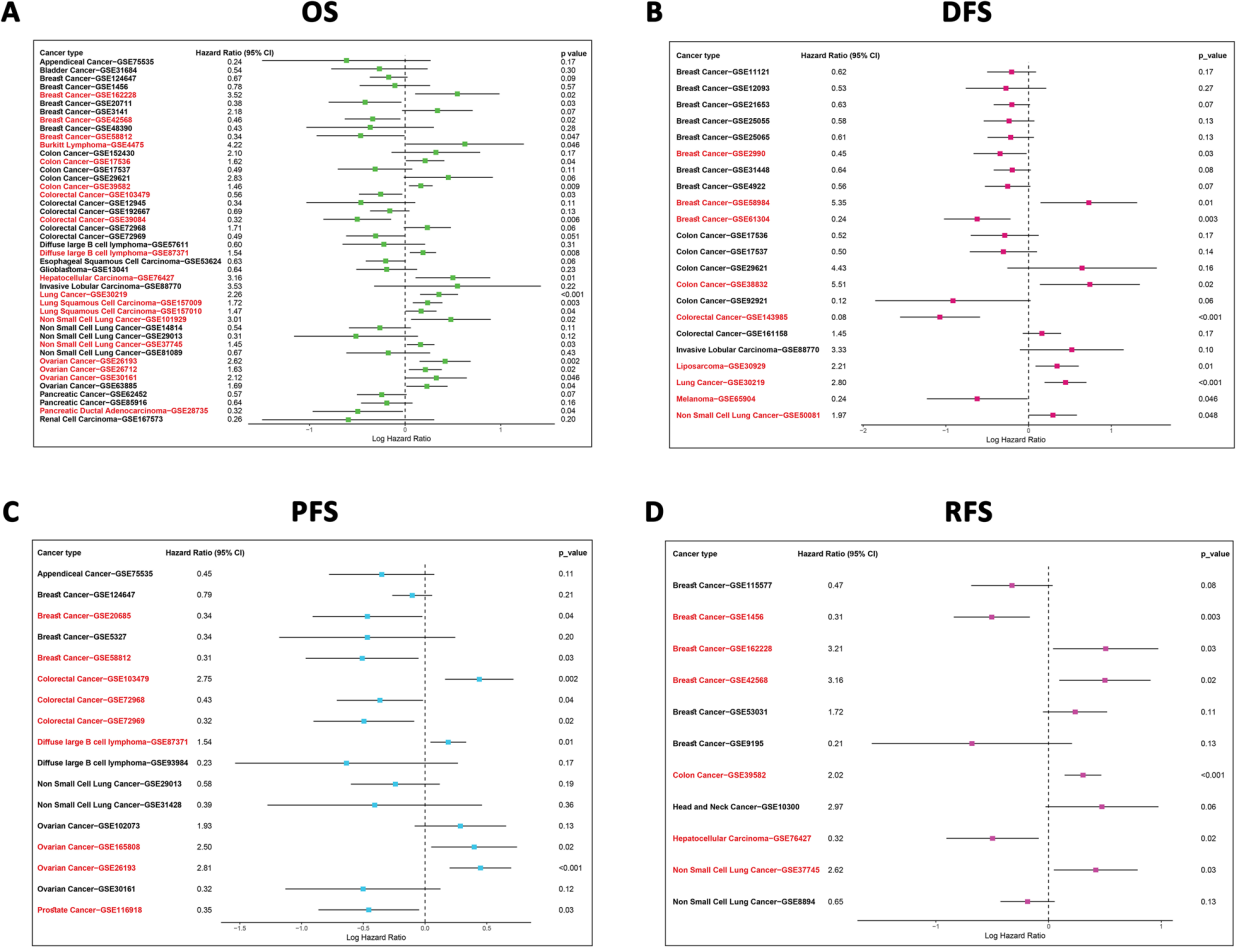
Forest plot representing univariate Cox regression analysis of HAND2-AS1. **A** Displays results of univariate Cox regression analysis of HAND2-AS1 for overall survival (OS), **B** for disease-free survival (DFS), **C** progression-free survival (PFS), and **D** for recurrence-free survival (RFS), in various microarray datasets. Red items signify statistical significance

We utilized a univariate Cox regression model to assess the relationship between HAND2-AS1 expression and OS, DFS, PFS, and RFS across diverse cancer types. Remarkably, increased HAND2-AS1 expression was observed to be significantly associated with unfavorable outcomes in various cancers. These included breast cancer, Burkitt lymphoma, colon cancer, CRC, hepatocellular carcinoma, diffuse large B cell lymphoma, lung cancer, lung squamous cell carcinoma, non-small cell lung cancer, ovarian cancer, and pancreatic ductal adenocarcinoma (Fig. 7A). Furthermore, with regard to DFS, higher HAND2-AS1 expression demonstrated a higher DFS rate in breast cancer, colon cancer, CRC, liposarcoma, lung cancer, melanoma, and non-small cell lung cancer (Fig. 7B). Conversely, in the context of PFS, an elevated HAND2-AS1 expression was significantly linked to reduced PFS rates in breast cancer, CRC, diffuse large B cell lymphoma, ovarian cancer, and pancreatic cancer (Fig. 7C). Interestingly, for RFS, elevated HAND2-AS1 expression was significantly associated with decreased PFS in breast cancer, colon cancer, hepatocellular carcinoma, and non-small cell lung cancer (Fig. 7D).

**Fig. 8:**
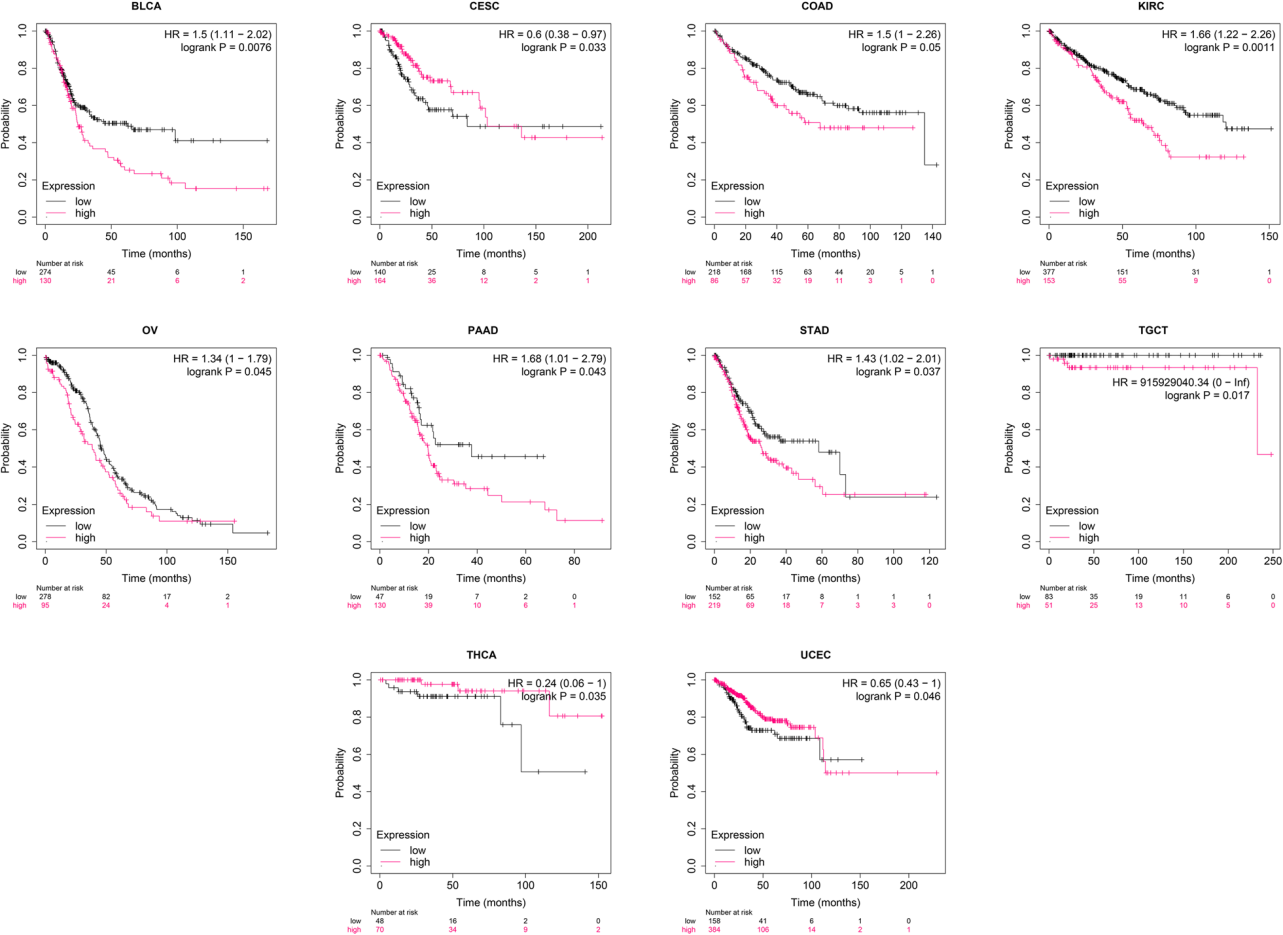
Kaplan–Meier plots demonstrating the relationship between HAND2-AS1 expression and overall survival (OS) in diverse cancers, highlighting significant outcomes. The pink line signifies high expression groups, while the black line corresponds to the low expression group

**Fig. 9:**
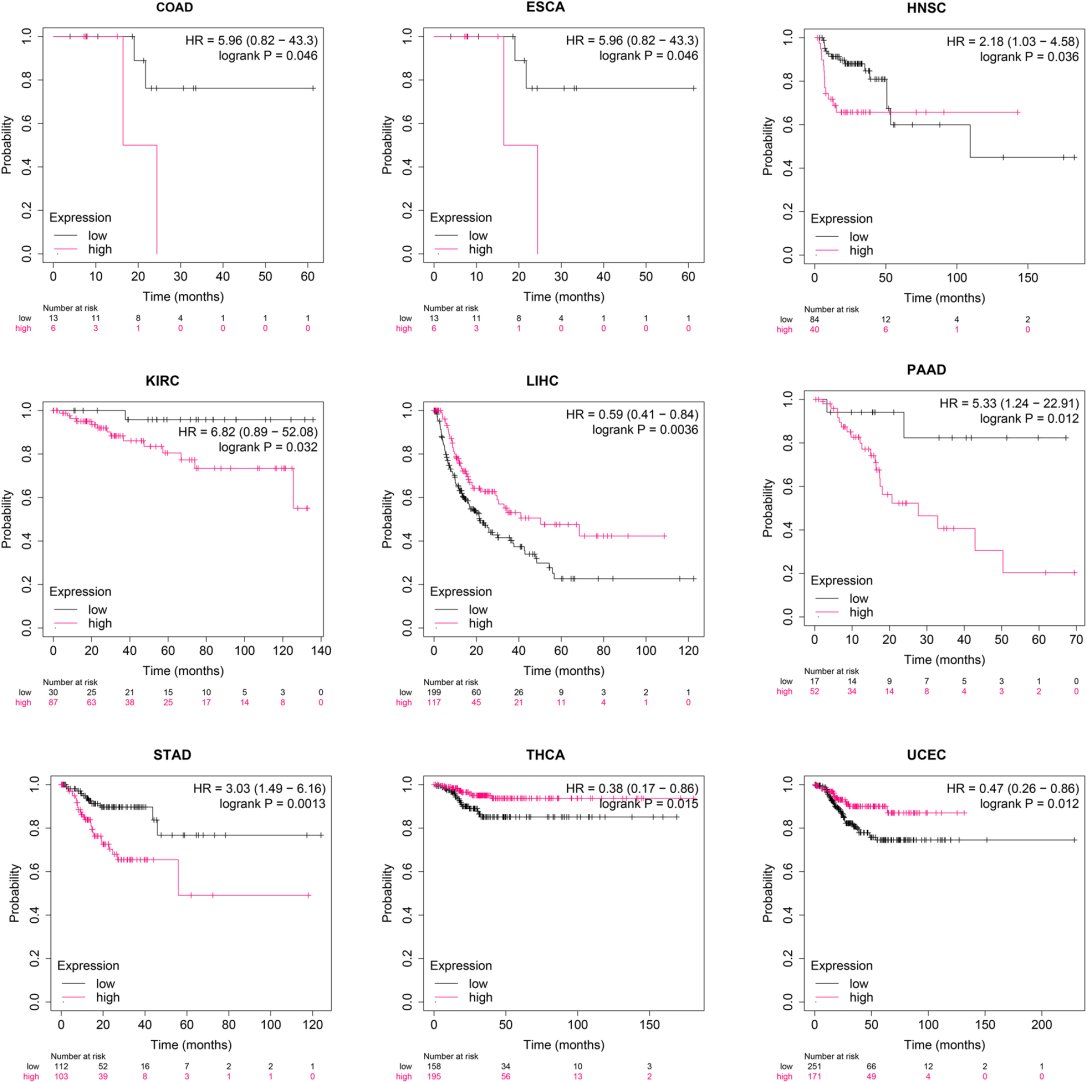
Kaplan–Meier analyses of RFS for HAND2-AS1, utilizing optimal cut-off values across different cancer patient groups. The pink line represents high expression groups, and the black line signifies the low expression group

Moreover, survival curves highlighted that lower HAND2-AS1 expression was indicative of notably worse OS (Fig. 8) and RFS (Fig. 9) time in ten and nine distinct cancer types, respectively. Taken together, these findings underscored the potential of HAND2-AS1 as a novel and valuable prognostic biomarker across multiple cancer.

### Immune cell infiltration analysis of HAND2-AS1 in pan-cancer

**Fig. 10:**
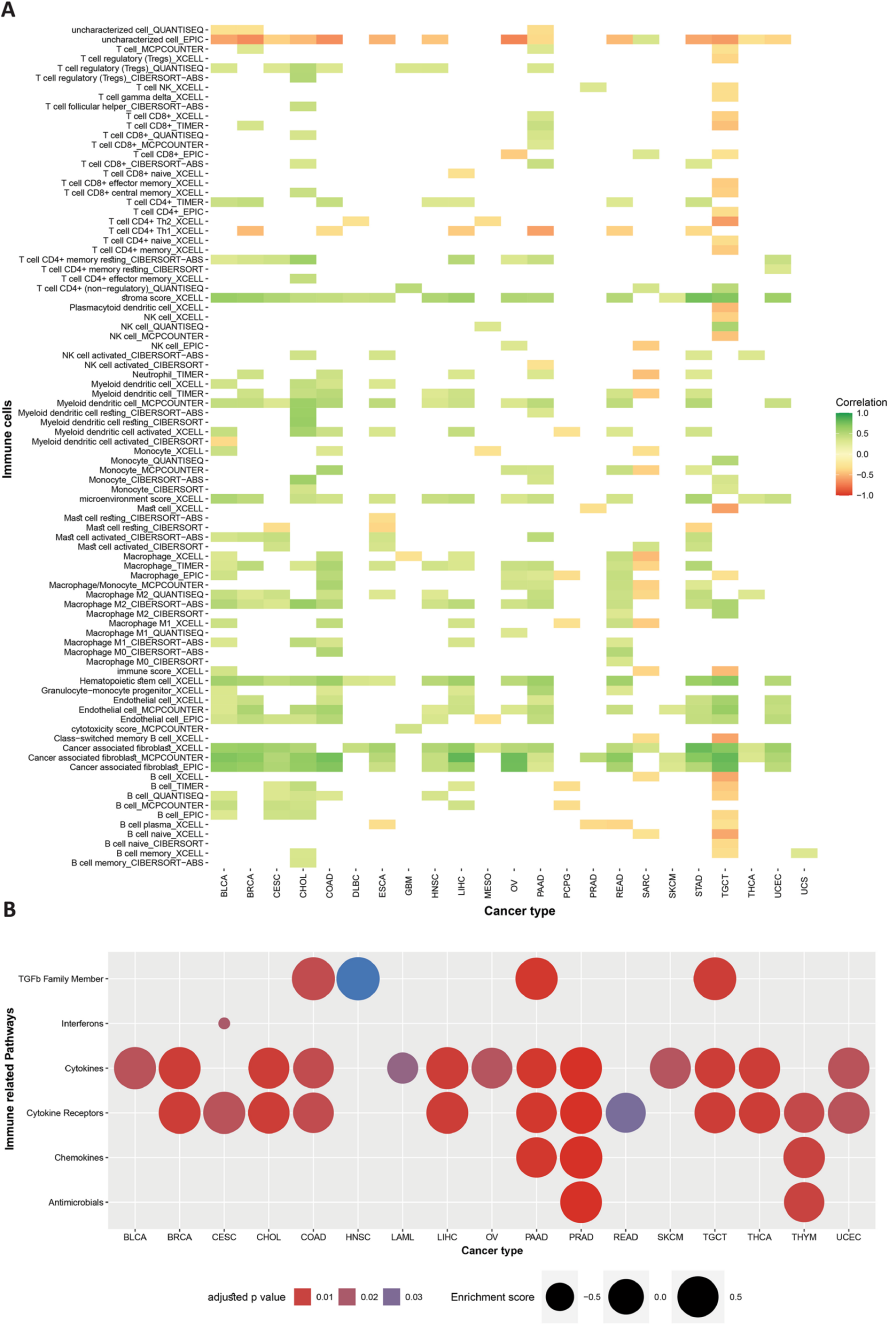
Explores the interplay between HAND2-AS1 and immune cell infiltration, as well as immune-related pathways. **A** Depicts the correlation between HAND2-AS1 expression and various immune cells from different algorithms. **B** Represents the relationship between HAND2-AS1 expression and diverse immune-related pathways

In order to explore the potential influence of HAND2-AS1 on the response of cancer patients to immunotherapy, a comprehensive analysis was conducted on immune-related data obtained from various algorithms. The analysis was carried out using the ggplot2 package in R. The results consistently revealed a positive correlation between the expression of HAND2-AS1 and the infiltration of various immune components. These immune components included tumor-associated macrophages (TAMs), natural killer (NK) cells, CD4+ T cells, CD8+ T cells, B cells, macrophages, mast cells, dendritic cells, among others. Notably, this correlation was observed across diverse cancers (Fig. 10A). Furthermore, additional data encompassing immune-related pathways, sourced from existing literature, were integrated into the analysis. This integration served to underscore the significant link between the expression of HAND2-AS1 and immune-related pathways. Notable pathways included those associated with the TGF-beta family, interferons, cytokines, and chemokines. These findings, presented in a pan-cancer context (Fig. 10B), collectively suggest that individuals with lower levels of HAND2-AS1 expression might exhibit a relatively immunosuppressive tumor microenvironment. This insight holds potential implications for the design and optimization of immunotherapeutic interventions.

### HAND2-AS1 DNA methylation analysis in pan-cancer

**Fig. 11:**
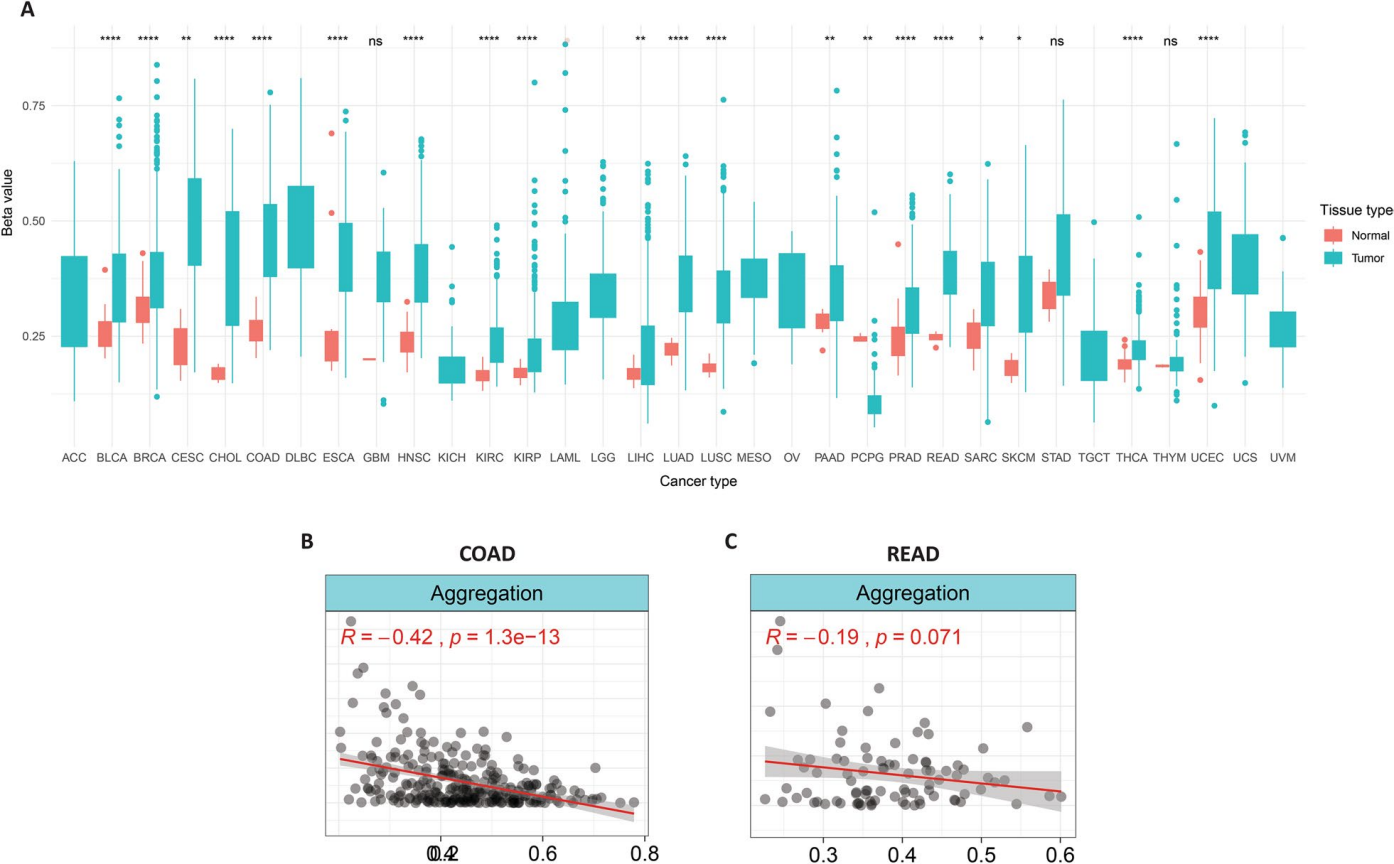
Analysis of HAND2-AS1 DNA methylation data from TCGA. **A** Illustrates the aggregated methylation value (beta-value) across all samples in the TCGA project. **B** Presents the correlation between gene expression and methylation value (beta-value) of HAND2-AS1 in TCGA-COAD-READ. *P < 0.05; **P < 0.01; ***P < 0.001; ****P < 0.0001

The HAND2-AS1 gene methylation data were obtained from the cBioPortal, which showed a substantial increase in the promoter methylation level of HAND2-AS1 in many cancers as depicted in Fig. 11A. Meanwhile, the DNA methylation pattern and HAND2-AS1 mRNA expression were negatively correlated in COAD and READ (Fig. 11B).

### Gene alteration of HAND2-AS1 in pan-cancer

**Fig. 12:**
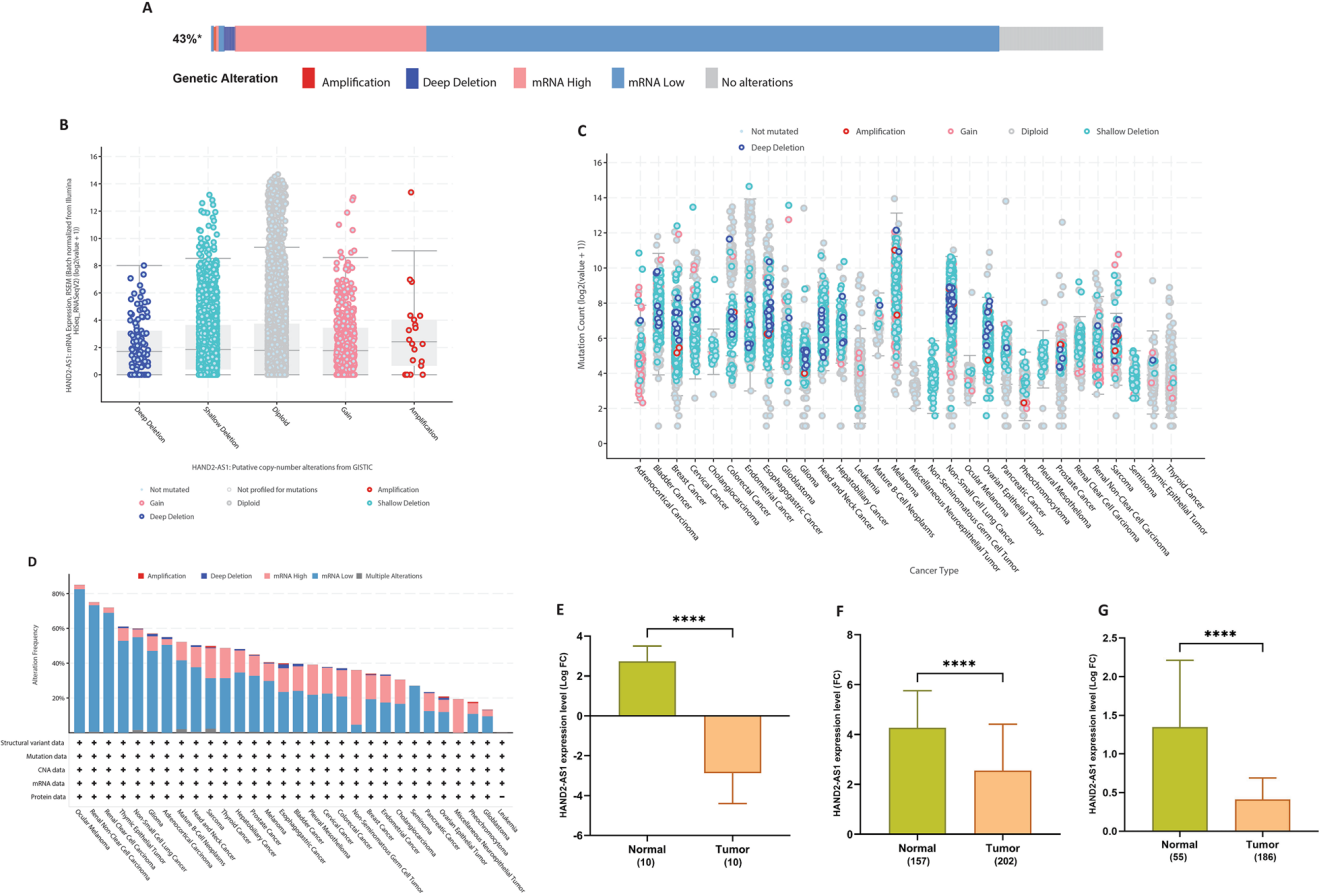
HAND2-AS1 mutation landscape and expression validation. **A** The relevance of different cancers and HAND2-AS1 expression where it is shown that mutations were mostly relevant to RNA expression. **B** The putative copy-number alterations from GISTIC of HAND2-AS1 in many TCGA cancers by the cBioPortal database. **C** Diagram of HAND2-AS1 mutations across cancer types. **D** mutational profile of HAND2-AS1 across different cancer types. **E** experimental validation based on 10 tumor and 10 adjacent normal tissues. **F**, **G** A set of more external validation based on homogenous GSE87211 (tumor = 202, normal = 157) and GSE68468 (tumor = 186, normal = 55) datasets, respectively

The intricate relationship between gene mutations, CNA, and tumor development is well acknowledged. In our investigation into HAND2-AS1 gene alterations, conducted through the cBioPortal platform, a distinct pattern emerged. Notably, the highest frequency of alterations in the HAND2-AS1 gene was observed among patients with ocular melanoma, renal non-clear and clear cell carcinoma. Within this context, the primary alteration type was marked by “mRNA low,“ followed by “mRNA high” alterations (Fig. 12A, D). Furthermore, a comprehensive exploration of the mutation frequencies of HAND2-AS1, characterized by significant deletions and amplifications, was carried out using the GISTIC database. This resource, renowned for its role in dissecting somatic mutations in human cancer, offered valuable insights into the landscape of HAND2-AS1 alterations (Fig. 12B). Across a range of cancers, a prevalent trend of HAND2-AS1 deletions was evident, accompanied by instances of gains and amplifications, albeit to a lesser extent (Fig. 12C). These findings contribute to our understanding of the genetic basis underlying tumor progression and provide potential avenues for further investigation and therapeutic exploration.

### HAND2-AS1 showed significant downregulation in CRC tissues

With regard to the emerging role of lncRNA HAND2-AS1 as a promising and innovative prognostic, immunomodulatory, and therapeutic biomarker in CRC, we conducted a comprehensive investigation of its expression level in CRC tissues (n = 10) and adjacent normal tissues (n = 10) using RT-qPCR. Our findings, illustrated in Fig. 12E, indicated a significant downregulation of HAND2-AS1 expression in CRC tissues compared to adjacent normal tissues (p = 1e−04). Furthermore, to validate this observation, we performed an external validation using samples from the microarray datasets, GSE87211 and GSE68468. This validation analysis (Fig. 12F, G) consistently demonstrated a substantial under-expression of HAND2-AS1 in CRC samples when compared to normal samples. Thus, in alignment with the TCGA expression analysis, our external validation further strengthened the fact that HAND2-AS1 is significantly downregulated in CRC tissues as opposed to normal tissues.

## Discussion

In recent decade, the exploration of molecular biomarkers implicated in the genesis and progression of human cancers has garnered substantial attention [22]. An area warranting deeper exploration involves the comparative analysis of precancerous stages and distinct cancer stages within healthy individuals [23]. Among the intricate regulatory mechanisms, lncRNAs have emerged as pivotal players, particularly in human diseases like cancer. Their modulation of mRNA stability and translation confers them with influential roles. Dysregulated expression of these non-coding RNAs exerts intricate effects on diverse facets of cancer cells, encompassing growth, proliferation, apoptosis, cell cycle control, invasiveness, metastasis, and drug resistance. Additionally, in the intricate network of cancer dynamics, lncRNAs can either function as tumor suppressors or oncogenes, orchestrating the modulation of their target mRNAs. This phenomenon is especially pronounced in CRC and other malignancies [24]. Based on our team’s previous studies aimed at identifying important biomarkers in CRC, we performed a comprehensive transcriptome analysis of lncRNA–mRNA interactions to identify further biomarkers that could aid in the diagnosis, treatment, and prognosis of this disease [25–27]. In this study, the analyses identified 2299 genes, and by identifying the important interactions of lncRNA–mRNA, the Co-expression and regulatory network of mRNA-lncRNA in CRC has been investigated. GO analysis demonstrated that DEGs were generally enriched in anatomical structure morphogenesis, as according to studies and based on the morphogenetic field escape control theory [28], tumors are formed when cells do not follow the normal patterning of the body, and cancer is part of an inevitable process where the organism lags behind in its performance [28, 29]. The GO analysis also revealed that cell-cell signaling, cell growth and connection, and regulation of ligand and receptor activity play significant roles in the development and progression of CRC. The KEGG analysis showed enrichment in tumor-related pathways, including calcium signaling pathway, protein digestion and absorption and cGMP-PKG signaling pathway, etc., whose potential mechanisms in the risk and pathogenesis of CRC have been shown in several studies [30–32]. As it has been stated in the studies that the change of intestinal microbiota, in addition to the effect on the development of cancer, can also be effective in preventing it. Changing the intestinal microbiota can also be effective in preventing CRC progression [33]. By analyzing the GO pathways associated with microbiota in the CRC group, Rui Huang and colleagues unveiled that the enriched entities predominantly contribute to processes such as protein digestion and absorption across immune, digestive, and endocrine systems. Furthermore, they identified an augmentation in secondary bile acid biosynthesis as well as an involvement in the adipocytokine signaling pathway [34]. Consequently, obvious alterations in the intestinal microflora among individuals diagnosed with CRC become evident following surgical resection and chemotherapy [34]. Also, about calcium signaling pathway, the interaction between cancer-associated fibroblasts (CAFs) and cancer cells is a complex signaling environment where cues from one cell compartment significantly impact the growth and survival of the entire niche. Recent studies have shown that Ca2 + signaling plays a significant role in promoting tumor growth and the development of CAF features [35]. Various mechanisms have been identified in the Ca2 + signaling between CAFs and cancer cells [36, 37]. The cGMP-PKG signaling pathway has also been investigated in several studies as a well-known and targeted therapeutic pathway in CRC [38, 39]. Furthermore, we identified the most important lncRNAs and mRNAs involved in CRC progression to perform downstream analyses. Our results showed that out of 22 final lncRNAs, HAND2-AS1, MIR100HG and MAGI2-AS3 are three important indicators in CRC, which affect the central and multiple DEGs of the network and can be used as new and potential predictors of DFS in CRC patients.

In result our study, HAND2-AS1 was identified as an important lncRNA that targets more than 90% of specific DEGs in the co-expression network. HAND2-AS1, located in proximity to HAND1, functions as an antisense lncRNA and is situated on chromosome 4q. Previous research has highlighted its potential as an oncogenic suppressor with a negative impact on metastasis [40–43]. In the findings of our current investigation, this lncRNA exhibited downregulation. Furthermore, the results from Kaplan–Meier analysis and multivariate survival assessment underscored the correlation between HAND2-AS1 expression and overall survival. Although functional studies on this lncRNA are primarily confined to other malignancies, they have demonstrated that heightened HAND2-AS1 expression curbs in vitro tumor cell proliferation and invasion, and dysregulated expression of this lncRNA impedes tumor migration in vivo [44, 45]. Regarding CRC, a study has shown role of HAND2-AS1. According to this study, HAND2-AS1 regulates KLF14 in CRC through the miR-1275 sponge [46]. In other words, KLF14 suppresses CRC progression by targeting the HAND2-AS1/miR-1275 axis. Also, they reported that the HAND2-AS1 rs2276941 polymorphism affects hsa-miR-1275 binding and is associated with an increased risk of colon cancer [46]. Furthermore, HAND2-AS1 regulates the miR-20 A/programmed cell death factor 4 axis to inhibit 5-fluorouracil resistance and promote cell apoptosis [47].

MIR100HG emerged as another significant lncRNA within the co-expression network, displaying upregulation. A study centered on understanding the impact of MIR100HG, a microRNA host gene situated on chromosome 11q24.1, investigated its role in metastasis and prognosis among individuals with cancer specifically CRC [48–51]. This investigation unveiled elevated MIR100HG expression in CRC tissues compared to normal mucosa, particularly in advanced CRC cases. Notably, patients exhibiting high MIR100HG expression faced compromised disease-free survival and overall survival in contrast to those with lower MIR100HG expression levels. Functional evaluations encompassing in vitro and in vivo assays unveiled that heightened MIR100HG expression facilitated CRC cell migration and invasion, alongside the formation of liver metastatic clusters in mouse models [52]. This study and others demonstrate results similar to ours, indicating that overexpression of MIR100HG may contribute to the progression of CRC and could be a potential therapeutic target and prognostic biomarker for patients with CRC [50, 52, 53]. Also, consistent with the theory that TGFβ triggers the expression of MIR100HG, which, in turn, increases TGFβ1 secretion, the analysis of human carcinomas by Panagiotis Papoutsoglou et al. [53] indicated that there was a correlation between MIR100HG expression and the expression of TGFB1, as well as its extracellular target TGFBI. Therefore, MIR100HG regulates the extent of TGFβ signaling by promoting TGFβ1 autoinduction and secretion in carcinomas [53].

MAGI2-AS3 is another significant lncRNA that, when overexpressed in tumor conditions [54–56], may indicate the activity and upregulation of high-risk genes [55]. In other words, positive regulation of MAGI2-AS3, like MIR100HG in CRC, could contribute to the development and progression of the disease. In same direction, in 2020, Xi Yang [57] and colleagues demonstrated that the MAGI2-AS3 rs7783388 polymorphism can increase the risk of CRC by affecting the binding affinity of the transcription factor GR to the MAGI2-AS3 promoter [57]. This finding adds to the growing body of evidence suggesting that single nucleotide polymorphisms in long noncoding RNAs may be involved in CRC susceptibility. Another study also showed that MAGI2-AS3 drives CRC progression through miR-3163/TMEM106B axis regulation [54].

## Conclusion

Our research notably underscores the prominence of HAND2-AS1, a lncRNA that surges to prominence not only within CRC but across a spectrum of cancers, indicating its far-reaching influence. Importantly, our exploration transcends CRC’s molecular landscape, uncovering HAND2-AS1’s expanded role as a significant participant in the complex pathways of cancer immunity. Altered methylation patterns and mutations across diverse cancer types implicate HAND2-AS1 as an instrumental force towards cancer progression. Its ability to mediate immune components and modulate immune-associated pathways echoes its potential to not only inform diagnostics but to wield influence over therapeutic strategies that harness the immune response against cancer. HAND2-AS1 emerges as an intricate key that unlocks not only insights into cancer development and progression but also possibilities for diagnostic precision and immune-based therapies.

### Supplementary Information

**Additional file 1: Table S1.** Significant DEGS and DE-lncRNAs. **Table S2.** Significant DEGS and DE-lncRNAs in the blue module. **Table S3.** Correlation of significant DEGS and DE-lncRNAs in the blue module. **Table S4.** Significant DEGS and DE-lncRNAs for the construction of regulatory network. **Table S5.** Significant GO term enriched for blue module. **Table S6.** Significant KEGG term enriched for blue module. **Table S7.** Significant GO term enriched for HAND2-AS1 targeted genes. **Table S8.** Significant KEGG term enriched for HAND2-AS1 targeted genes.

## Declarations

### Availability of supporting data

All the data employed in this study are comprehensively presented within the article, and availability upon reasonable request from the corresponding author is ensured. The relevant datasets can also be accessed through direct web links: TCGA: https://www.cancer.gov/ ; GTEx: https://commonfund.nih.gov/GTEx/ ; GDC: https://gdc.cancer.gov/ ; cBioPortal: https://www.cbioportal.org/ ; Kaplan–Meier Plotter: http://www.kmplot.com/analysis/ ; DAVID: https://david.ncifcrf.gov/ ; TIMER2.0: http://timer.cistrome.org/ ; Smartapp: www.bioinfo-zs.com/smartapp/; TISDB: http://cis.hku.hk/TISIDB/index.php.
